# The role of mitochondria-associated ER membranes in disease pathology: protein complex and therapeutic targets

**DOI:** 10.3389/fcell.2025.1629568

**Published:** 2025-06-30

**Authors:** Jin Zhang, Dan Li, Lingjie Zhou, Yanying Li, Qian Xi, Liuyun Zhang, Juan Zhang

**Affiliations:** ^1^ School of Medicine, University of Electronic Science and Technology of China, Chengdu, Sichuan, China; ^2^ Department of Laboratory Medicine, Sichuan Provincial Key Laboratory for Human Disease Gene Study, Sichuan Provincial People’s Hospital, School of Medicine, University of Electronic Science and Technology of China, Chengdu, Sichuan, China; ^3^ School of Laboratory Medicine, Chengdu Medical College, Chengdu, China

**Keywords:** mitochondria-associated ER membranes, mitochondria, endoplasmic reticulum, cancer, neurodegeneration, diabetes mellitus, cardiovascular diseases

## Abstract

The dynamic interactions among organelles play a crucial role in facilitating various intercellular functions, with the interaction between the endoplasmic reticulum (ER) and mitochondria being acknowledged as a prominent example of an interorganellar system. Numerous studies have established that the majority of proteins located at the physically tethered regions between the mitochondria and ER, referred to as mitochondria-associated ER membranes (MAMs), play a crucial role in intracellular physiological processes. MAMs are dynamic membrane coupling regions arising from the interaction between the ER and the outer mitochondrial membrane (OMM). MAMs regulate many biological processes, such as Ca^2+^ transport, lipid metabolism, and mitochondrial dynamics. A recent study has demonstrated that the proteins associated with MAMs are crucial for both the structural integrity and functional capabilities of the MAMs. Dysregulations in the MAMs proteins are implicated in the onset and progression of various associated diseases, including cancer, neurodegenerative disorders, diabetes mellitus, and cardiovascular diseases. In this review, we provide a comprehensive overview of the protein complex associated with MAMs. We examine its involvement in the pathological mechanisms underlying these diseases, focusing on its functional roles. Additionally, we evaluate and consider the potential of MAMs as therapeutic targets for these diseases.

## Graphical Abstract

**Figure:**
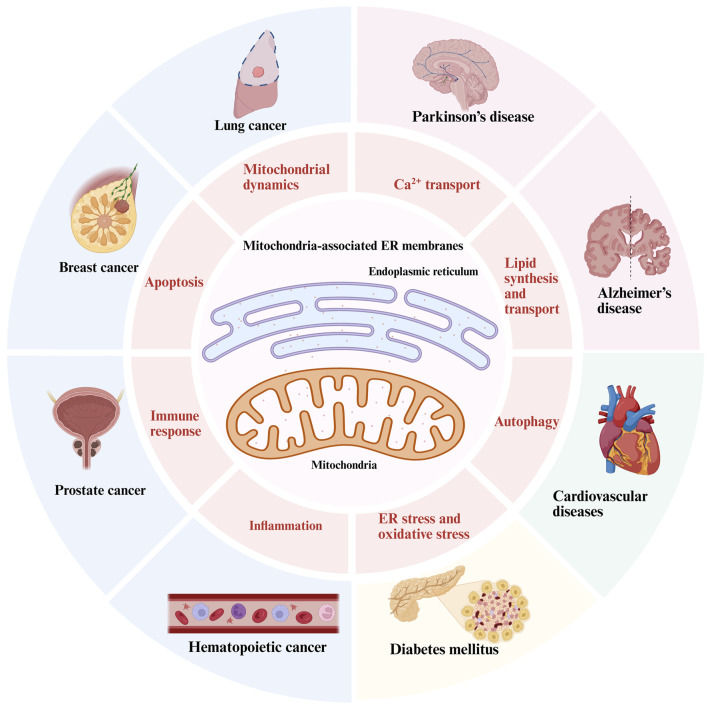
The relationship between MAMs’ cellular physiology and diseases.

## 1 Introduction

As is widely recognized that organelles operate not as independent units but rather in a networked manner, where their functionality is influenced by interactions with other organelles through membrane contact sites (Loncke et al., 2021). These contacts facilitate the formation of microdomains that encompass various cellular functions by serving as platforms for signaling complexes and supporting specific biological processes. Among interorganellar networks, MAMs are recognized as communication platforms that facilitate bidirectional regulation of cellular physiological functions between the mitochondria and the ER, indirectly impacting cellular homeostasis and function. The involvement encompasses various processes, including Ca^2+^ transport, lipid metabolism, and mitochondrial dynamics, among others (Wu et al., 2023).

**FIGURE 1:**
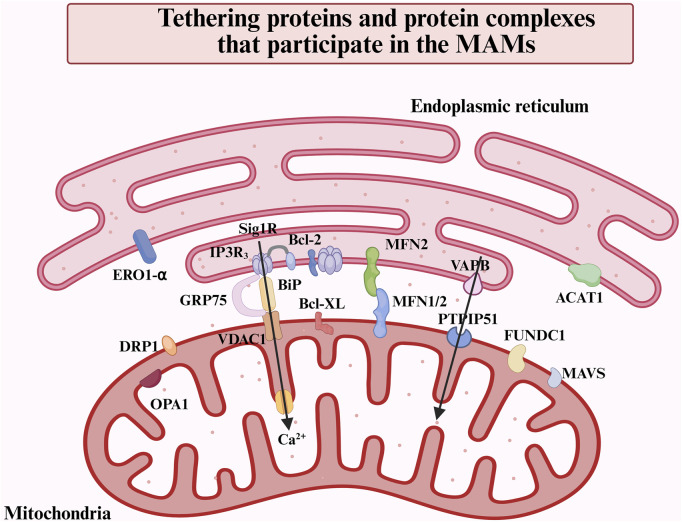
Overview of the main proteins and protein complexes in the MAMs. MAMs are defined as regions where the OMM and specific regions of the ER membrane overlap without membrane fusion. This structural arrangement facilitates inter-organelle communication and bi-directionally modulates cellular functions via tethering proteins. The major proteins and protein complexes on MAMs include IP_3_R-GRP75-VDAC1, VAPB-PTPIP51, MFN2-MFN1/2, OPA1, DRP1, FUNDC1, MAVS, ACAT1, etc.

The MAMs are defined as regions in which the OMM and specific regions of the ER membrane coexist in proximity without undergoing membrane fusion (Wang N. et al., 2021). Utilizing electron microscopy, it has been observed that the intermembrane distance between the ER and the OMM is mainly maintained within a range of 10–80 nm (Csordás et al., 2006). From a morphological perspective, the characteristics of MAMs can be defined by their lateral dimensions and the distance between gaps, which are predominantly influenced by the specific selection of membrane proteins in the local environment. Various proteomic analyses of the structure of MAMs have identified a total of 991 or 1212 distinct proteins present within these membranes (Wang N. et al., 2021). Included among these were widely acknowledged MAMs proteins and protein complexes: the IP_3_Rs-GRP75-VDACs complex, the VAPB-PTPIP51 complex, the MFN2/1-MFN2 complex, Sig-1R, DRP1, FUNDC1, OPA1, MAVS, ERO1-α, ACAT1(Figure 1). And the mass spectrometry analysis classified the constituent proteins into three distinct categories: proteins that are exclusively found in MAMs; proteins that are present in both MAMs and other organelle structures; and proteins that are transiently associated with MAMs (Wang N. et al., 2021). Furthermore, the MAMs proteins can be categorized based on their functional roles, which encompass Ca^2+^ regulatory proteins, lipid synthesis and transport proteins, redox regulatory proteins, as well as autophagy-related proteins, among others (Li Y. E. et al., 2022).

The disruption of MAMs homeostasis is characterized by Ca2+ overload and the accumulation of unfolded or misfolded proteins, among other factors. This dysregulation ultimately results in compromised or complete loss of MAMs functionality, which can precipitate cellular degeneration and apoptosis. Impairments in the structural integrity and function of MAMs may contribute to the onset of various pathological conditions and diseases (Ding et al., 2024). Given that the mechanisms governed by MAMs are intricately linked to cell fate, such as metabolism, invasion, metastasis, and apoptosis, pertinent research has commenced to investigate and develop various therapeutic strategies targeting MAMs (Wu et al., 2023). This review examines the mechanisms of MAMs in regulating cellular processes and influencing cell fate via its vital functions. In addition, we explore how the dysregulation of MAMs contributes to pathophysiological conditions and various disease processes, with particular emphasis on potential strategies for targeted therapeutic interventions.

## 2 The basic functions of the MAMs

Stable interactions in the MAMs proteins facilitate the integration of various cellular functions performed by organelles. The mitochondria and ER collaboratively regulate several cellular biological processes by modulating the proteins on the MAMs. In the subsequent discussion, we will elucidate the fundamental cellular functions of MAMs, beginning with the MAMs proteins (Table 1).

**TABLE 1:** Multifaceted role of MAMs: summary of the most important functional roles of some MAMs-resident proteins mentioned in this review.

Principal functions	MAM resident proteins	Relevant functions in MAMs	References
Ca^2+^ transportation	IP_3_R	Regulating ER-Ca^2+^ release to mitochondria; Interacting with VDAC via GRP75	Szabadkai et al. (2006)
	MCU	Taking part in the transport of Ca^2+^ into the mitochondrial matrix	Liu et al. (2020a)
	Sig-1R	Interacting with IP_3_R; Play important roles in Ca^2+^ transfer to mitochondria and MAMs stability	Hayashi and Su (2007)
	GRP75	Linking with IP_3_R and VDAC in MAMs	Gu et al. (2020)
	DJ-1	Interact and comigrate with the IP_3_R3-Grp75-VDAC1 complex	Liu et al. (2019)
	VDAC1	Localizing in OMM involved in Ca^2+^ transport to mitochondria	Liu et al. (2019)
Lipid synthesis	ACAT1	Regulating cholesterol metabolism	Puglielli et al. (2001)
	ORP5/8	Connecting to and collaborating with the MIB/MICOS complexes to mediate non-vesicular transport of PS from the ER to mitochondria	Monteiro-Cardoso et al. (2022)
	PSS1/2	Playing role in PS synthesis and trafficking	Stone and Vance (2000), Filadi et al. (2016)
	PISD	Playing role in PE synthesis	Aaltonen et al. (2016)
	PEMT2	Playing role in PC synthesis	Rowland and Voeltz (2012)
Mitochondrial dynamics	MFN1	Tethering mitochondria to ER through connection with ER-located MFN2	De Brito and Scorrano (2008)
	MFN2	Forming hetero- or homodimers with MFN1/2; Responding to ER stress	Naón et al. (2023)
	DRP1	Playing role in mitochondrial fission; Interacting with MFF	Lee et al. (2016)
	OPA1	Controlling mitochondrial dynamics, cristae integrity, energetics and mtDNA maintenance	Mishra et al. (2014)
	MFF	Binding to AMPK under sustained stress, coordinating mitochondrial division	Otera et al. (2010)
	MIRO1/2	Involving in mitochondrial motility	Lahiri and Klionsky (2017)
Autophagy	ATG5/ATG14	Inducing autophagosome formation from MAMs under starvation; Interacting with Beclin 1	Hamasaki et al. (2013)
	PTPIP51	Playing role in Ca^2+^ transfer from ER to mitochondria, in lipid transfer; Interacting with VAPB	Gomez-Suaga et al. (2017)
	VAPB	Interacting with PTPIP51	Zhao et al. (2018)
	FUNDC1	Accumulating at ER-mitochondrial contacts by interacting with calnexin under hypoxic stress and recruit DRP1 to the MAMs	Wang et al. (2021a)
	PINK1	Interacting with Parkin, experiencing a phosphorylation modification	Gelmetti et al. (2017)
	Parkin	Experiencing ubiquitination modification, which subsequently facilitate the process of autophagy	Quinn et al. (2020)
	Beclin 1	Inducing autophagosome formation from MAMs and interacting with Bcl-2 and IP_3_R	Gelmetti et al. (2017)
	PML	Recruiting PP2A to the IP_3_R3-PKB complex	Pedriali et al. (2017)
ER stress and oxidative stress	ATF6	Interacting with VAPB to directly inhibit UPR response	Glembotski et al. (2020)
	PERK	Engaging with MFN2 to enhance the contact and facilitating apoptosis induced by mitochondrial ROS	Smedley et al. (2021)
	IRE1	Increasing the abundance and stability of IP_3_R, meditating ER-mitochondria dynamics	Carreras-Sureda et al. (2019)
	ERO1	Activating Ca^2+^ release from IPRs during ER stress	Li et al. (2009)
Inflammation	NLRP3	Assembling the inflammasome, triggering an inflammatory response	Kelley et al. (2019)
Immune response	MAVS	Combining RIG-I/MDA5 to transmits viral RNA recognition signals and activating the NF-κB and IRF3 pathways	Rajput et al. (2011)
Apoptosis	Fis1	Regulating ER-mitochondria tethering, apoptosis, and mitochondrial dynamics	Iwasawa et al. (2011)
	Bap31	Promoting Ca^2+^ release from ER	Iwasawa et al. (2011), Cho et al. (2017)
	Bcl-xl	Preventing intracellular Ca^2+^ overload and apoptosis	Williams et al. (2016)
	Bcl-2	Binding to and inhibiting excessive, pro-apoptotic Ca^2+^ release from IP_3_Rs	Hanson et al. (2008)
	Bax	Promoting the release of cytochrome c and facilitating apoptosis	Lee et al. (2014)

### 2.1 Ca^2+^ transport

The primary signal conveyed by MAMs is Ca^2+^. As a crucial second messenger, Ca^2+^ plays a vital role in cellular functions and the maintenance of cell viability. Intracellular Ca^2+^ is predominantly sequestered within the ER, where its concentration significantly exceeds that found in the cytosol and mitochondria (Jiang et al., 2023). As a significant site for Ca^2+^ transportation, MAMs is essential for the translocation of Ca^2+^ from the ER to the mitochondria (Prinz et al., 2020).

**FIGURE 2:**
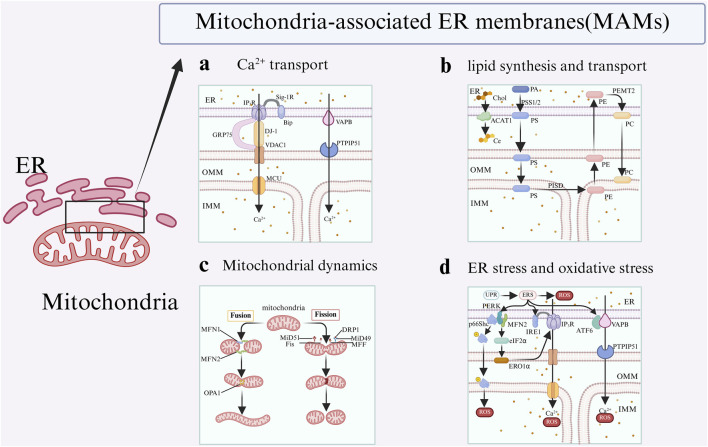
Key functions of MAMs. The representative related proteins of MAMs are involved in four functions: a: MAMs regulate Ca^2+^ flux between the ER and the mitochondria; b: MAMs facilitate lipid svnthesis and transport; c: MAMs regulate mitochondrial dynanic, including fusion,fission; d: MAMs modulate ER stress and oxidative stress.

It is widely accepted that Ca^2+^ are translocated through a protein complex that includes inositol 1,4,5-trisphosphate receptors (IP_3_Rs) located on the ER, as well as the glucose-regulated protein of 75 kDa (GRP75) and voltage-dependent anion channel 1 (VDAC1) situated on the mitochondria (Szabadkai et al., 2006; Giorgi et al., 2018). Subsequently, Ca^2+^ is translocated into the mitochondrial matrix from OMM via the mitochondrial calcium uniporter (MCU). Consequently, the IP_3_R-GRP75-VDAC1-MCU axis constitutes a Ca^2+^ transfer pathway between the ER and mitochondria, which is regulated by MAMs. In a recent study, DJ-1 was also shown to be present in MAMs and could interact with IP_3_R-GRP75-VDAC proteins complex and become an essential component (Basso et al., 2020) (Figure 2a). Secondly, mitofusin 2 (MFN2), which is situated on both the ER surface and the OMM, is classified as a resident protein in MAMs. It serves dual functions as a critical mediator of mitochondrial fusion and as a facilitator of the interaction between IP_3_R and VDAC1 (De Brito and Scorrano, 2008; Filadi et al., 2015). Furthermore, Sigma-1 receptor (Sig-1R) is recognized as a marker of the MAMs, which facilitates the extension of Ca^2+^ signaling from the ER to the mitochondria. After the activation of IP_3_Rs, Sig-1R associates with IP_3_R3 and dissociates from BiP. This interaction serves to stabilize IP_3_R3 at the MAMs, thereby facilitating enhanced Ca^2+^ fluxes to the mitochondria mediated by IP_3_R3. Moreover, elevated levels of mitochondrial Ca^2+^ enhanced the production of NADH via enzymes involved in the tricarboxylic acid (TCA) cycle, thereby facilitating an increase in ATP synthesis and the generation of reactive oxygen species (ROS) (Rizzuto et al., 2012). Therefore, the Ca^2+^ transportation, regulated by MAMs, is both associated with mitochondrial respiration and energy metabolism.

However, Ca^2+^ functions as a double-edged sword, possessing the capacity to enhance metabolic processes while simultaneously triggering cellular apoptosis. Sustained accumulation of Ca^2+^ within mitochondria has detrimental effects on cellular viability. An overabundance of mitochondrial Ca^2+^ uptake initiates the opening of the mitochondrial permeability transition pore (mPTP), which subsequently leads to the release of cytochrome c. This sequence of events results in mitochondrial dysfunction and ultimately culminates in cellular apoptosis (Giorgi et al., 2018; Chen et al., 2023; Jiang et al., 2023).

Owing to the significance of Ca^2+^ in cellular process, the level of Ca^2+^ in cells is strictly controlled. And further, altered Ca^2+^ homeostasis may lead to different pathological conditions, depending on what kind of cell involved (Liu Y. et al., 2020). Collectively, MAMs-mediated Ca^2+^ homeostasis has robust connection with various cellular processes, which is a central hub in the pathogenesis of cancers, neurodegenerative disorders, and cardiovascular diseases and so on.

### 2.2 Lipid synthesis and transport

It has been reported that, alongside vesicular transport, lipid transport between organelles may transpire via tight junctions or through protein-mediated interactions between membranes (Vance, 2020). The proteins that are characteristic of MAMs promote the lipid synthesis and transport in the MAMs via the later (Anastasia et al., 2021).

The synthesis of lipids in the MAMs necessitates the participation of associated enzymes situated on the membranes of both organelles. Furthermore, various intermediates involved in this process are exchanged repeatedly in the MAMs (Jiang et al., 2023). For example, in the ER, phosphatidic acid (PA) is synthesized through the action of the enzyme phosphatidylserine synthases 1 and 2 (PSS1 and PSS2), which convert it into phosphatidylserine (PS). Subsequently, PS is transported to the mitochondria, where it undergoes conversion into phosphatidylethanolamine (PE) via the enzymatic activity of phosphatidylserine decarboxylase (PISD) located on the inner mitochondrial membrane (IMM). Ultimately, PE is transported back to the ER, where it undergoes conversion to phosphatidylcholine (PC) through the action of the enzyme PEMT2. Subsequently, the PC is returned from the ER to the mitochondria (Janikiewicz et al., 2018) (Figure 2b **)**.

Additionally, enzymes involved in cholesterol biosynthesis are localized within MAMs. the MAMs fraction exhibits an enrichment of cholesterol and ceramides, particularly in hepatocytes (Csordás et al., 2018). ACAT1, present in MAMs, plays a crucial role in the esterification of intracellular free cholesterol into cholesterol esters. This process is essential for maintaining the balance between bound and free cholesterol in resting states (Shi et al., 2021). Research indicates that modifications in cholesterol levels can influence the interaction between the ER and mitochondria. Specifically, a reduction in cellular cholesterol levels leads to an enhanced connection between the mitochondria and the ER (Fujimoto et al., 2012). Consequently, MAMs’ lipid and proteins play an important role in determining mitochondrial lipid composition and facilitating lipid transport among organelles. And the dysregulated lipid synthesis and transport will influence lipid homeostasis and MAMs’ functions, which exert important impact on various diseases.

### 2.3 Mitochondrial dynamics

Mitochondrial dynamics encompasses the processes of fusion, fission, and transport, which are essential for the proper functioning of signal transduction and metabolic pathways (Chen et al., 2023).

Mitochondria are semi-autonomous organelles harboring their own DNA (mtDNA) and require continuous repair and renewal of components to sustain functionality. The regulation of mitochondrial dynamics is crucial for the optimal functioning of mitochondria and the determination of cellular outcomes (Tilokani et al., 2018). Mitochondrial dynamics facilitate the removal of damaged components from mitochondria, and dysfunctional mitochondria can be completely eliminated through the process of mitophagy, thereby mitigating the risk of additional cellular damage (Youle and van der Bliek, 2012). In a certain sense, mitophagy can be considered a component of mitochondrial dynamics. In detail, fission is essential for mitochondrial quality control as it enables the elimination of damaged or dysfunctional mitochondria. Additionally, it contributes to the induction of apoptosis in response to substantial cellular stress. In contrast, mitochondrial fusion promotes the mixing and exchange of intramitochondrial components among mitochondria, thereby playing a crucial role in the preservation of mitochondrial functionality (Youle and van der Bliek, 2012).

Research has demonstrated that MAMs play an important role in regulating mitochondrial morphology, including the size, shape, and length, by maintaining the balance between mitochondrial fission and fusion processes (Scorrano, 2013) (Figure 2c). Mitochondrial fusion requires the coordinated merging of both the OMM and IMM, a process that is predominantly regulated by three GTPases.: mitofusin 1 (MFN1), MFN2, and optic atrophy type 1 (OPA1) (Vezza et al., 2022). The fusion of the OMM is facilitated by the proteins MFN1 and MFN2 (Chen et al., 2003; Detmer and Chan, 2007), while the fusion of the inner mitochondrial membrane is governed by OPA1 (Al Ojaimi et al., 2022). MFN1 and MFN2 are located on the OMM and are instrumental in the fusion process of mitochondria, resulting in the establishment of interconnected mitochondrial networks (Filadi et al., 2018). It is noteworthy that MFN2 is the first protein identified as directly facilitating the tethering of the ER to mitochondria in mammals (De Brito and Scorrano, 2008), serving as a crucial regulator of ER-mitochondrial contact site tethering (Tilokani et al., 2018). In addition to its presence in the OMM, MFN2 is also localized at MAMs (Filadi et al., 2018; Hu et al., 2021). Within MAMs, MFN2 facilitates the interaction between the ER and mitochondria by forming physical associations with either MFN1 or MFN2 situated on the OMM (Naon et al., 2016). Research has demonstrated that the deletion of MFN2 in murine fibroblasts results in the disruption of ER-mitochondrial contact sites, consequently leading to an increased spatial separation in the MAMs (De Brito and Scorrano, 2008).

The ER exhibits significant interaction with mitochondria, particularly in its role in facilitating mitochondrial fission through the encapsulation of mitochondrial segments. This observation implies that MAMs play a crucial role in the process of mitochondrial fission (Friedman et al., 2011; Jiang et al., 2023). Mitochondrial fission is initiated at locations where ER tubules exert constrictive forces on the mitochondria (Friedman et al., 2011). Research has demonstrated that proteins located on MAMs play an important role in the regulation of mitochondrial division. The primary proteins include fission protein 1 (Fis1) (Losón et al., 2013), MiD49 and MiD51 (Palmer et al., 2013), mitochondrial fission factor (MFF) (Otera et al., 2010), dynamin-related protein 1 (DRP1) (Smirnova et al., 2001). These proteins are recruited to the contact sites in the MAMs, where they facilitate the constriction of mitochondrial membranes, thereby promoting the division process (Lee et al., 2016).

Typically, to enhance mitochondrial function, mitochondria regulate a homeostatic equilibrium between the processes of fusion and fission (Wu et al., 2023). In contrast, dysregulated mitochondrial dynamics are linked to various diseases that exhibit deficiencies in mitochondrial function and abnormal cellular responses.

### 2.4 Autophagy

Autophagy is a meticulously regulated biological process that facilitates the degradation and recycling of misfolded proteins and damaged organelles, thereby contributing to the maintenance of cellular homeostasis (Mizushima et al., 2011). As a core mechanism for maintaining cellular homeostasis, autophagy encapsulates abnormal components within a double-membrane structure known as autophagosomes and achieves nutrients recycling through lysosomal degradation (Mizushima et al., 2011). This process involves multiple physiological functions, including protein quality control and stress response (Ichimiya et al., 2020).

Empirical studies have shown that the integrity of MAMs is crucial for the process of autophagosome formation (Li Y. E. et al., 2022). The interaction between the ER protein VAPB and the mitochondrial protein PTPIP51 regulates Ca^2+^ transport between the ER and mitochondria. Moreover, when any protein is absent, the interaction between IP_3_R and VDAC decreases, leading to impaired mitochondrial Ca^2+^ uptake, which in turn activates autophagy (Gomez-Suaga et al., 2017). Additionally, research has indicated that vesicle-associated membrane protein-associated proteins (VAPs), including VAPA and VAPB, enhance autophagy by modulating the interactions between the ER and other organelles or by promoting the formation of MAMs, as well as by directly engaging with autophagy-related (ATG) proteins (Zhao et al., 2018). This suggests that different tethering proteins may differentially regulate the formation of autophagosomes and the specific structures targeted for autophagic degradation.

As a typical representative of selective autophagy, mitophagy maintains cellular homeostasis by eliminating dysfunctional or excess mitochondria, preventing abnormal metabolism and apoptosis (Poole and Macleod, 2021). Its core mechanism involves two MAMs-related pathways. Firstly, under hypoxic conditions, the OMM protein FUN14 domain containing 1 (FUNDC1) localizes to MAMs by interacting with the ER membrane protein calnexin (Wu et al., 2016a). As mitophagy is initiated, the dissociation of FUNDC1 from calnexin allows it to recruit DRP1 to MAMs, driving mitochondrial fission (Wu et al., 2016b). Subsequently, when autophagy is activated, the PINK1/Parkin complex accumulates at MAMs, promoting the formation of autophagosomes by enhancing ER-mitochondria interactions. These two pathways together reveal the important role of MAMs as a hub for mitochondrial quality control, with their dynamic restructuring directly determining the process and efficiency of mitophagy.

### 2.5 ER stress and oxidative stress

When the protein folding demand of the ER exceeds its capacity, unfolded or misfolded proteins accumulate in the lumen, triggering ER stress and activating the unfolded protein response (UPR) (Fan and Simmen, 2019; Qin et al., 2023). The initiation of the UPR is characterized by the activation of three ER transmembrane proteins: activating transcription factor 6 (ATF6), protein kinase R-like endoplasmic reticulum kinase (PERK), and inositol requiring enzyme 1 (IRE1) and inter-organelle communication facilitated by MAMs (Figure 2d).

Initially, ER stress can induce a perinuclear redistribution of both ER and mitochondrial networks, resulting in an increase in ER-mitochondrial contacts (Fan and Simmen, 2019). MAMs-enriched molecular chaperones, including GRP78 and calnexin, are directly involved in UPR signaling (Gutiérrez and Simmen, 2018), while Ca^2+^ signaling regulatory proteins such as GRP75 and MFN2 participate in stress adaptation by maintaining Ca^2+^ homeostasis (Yuan et al., 2020) and regulating the PERK pathway (Muñoz et al., 2013). Notably, all core UPR sensors are localized in MAMs, creating a unique regulatory microenvironment. The presence of IRE1 promotes ATP production by regulating IP_3_R-mediated mitochondrial Ca^2+^ influx (Carreras-Sureda et al., 2019). Furthermore, the ubiquitination of IRE1α inhibits apoptosis induced by ER stress (Chern et al., 2019). The PERK associates with MFN2 to augment the connection between MAMs and to mediate mitochondrial ROS-induced apoptosis (Muñoz et al., 2013). Upon depletion of MFN2, the activation of PERK initiates ER stress signaling via the PERK-eIF2α-ATF4-CHOP pathway (Liu X. et al., 2021). Additionally, ATF6 extends cellular lifespan by modulating Ca^2+^ transfer and signaling via indirectly inhibiting the interaction between ERS-induced VAPB and PTPIP51(De Vos et al., 2012; Zhang et al., 2020). Moreover, VAPB overexpression inhibits ATF6 transcription, blocking the UPR response (Gkogkas et al., 2008).

MAMs maintains cellular communication between the mitochondria and ER by the hydrogen peroxide (H_2_O_2_) nanodomains, producing the ROS, then influencing Ca^2+^ signaling and mitochondrial functionality (Booth et al., 2016). Endoplasmic reticulum oxidoreductin 1-α (ERO1) and p66Shc are two key proteins which come from MAMs that can directly produce ROS. ERO1 elevated expression enhances ROS production by facilitating Ca^2+^ release from IP_3_Rs (Anelli et al., 2012). Conversely, p66Shc associated with ROS generation, undergoes phosphorylation at Ser36 under oxidative stress, leading to isomerization and subsequent dephosphorylation, ultimately resulting in its translocation to MAMs to mediate ROS production (Pinton et al., 2007).

Moderate activation of ER stress and oxidative stress can enhance cellular adaptability, but sustained stress leads to a vicious cycle that promotes disease progression. Targeting the intersection of the two may provide new therapeutic strategies for cancer, neurodegenerative diseases, and diabetes mellitus.

### 2.6 Inflammation

Inflammation functions as an essential immune defense mechanism in living organisms, reacting to abnormal pathological stimuli including ischemia, heightened immune responses. Meantime, it is also acknowledged as a major pathological mechanism contributing to a range of conditions, including cardiovascular diseases, cancer, and metabolic disorders. Research has confirmed that the MAMs participates in the inflammatory response by regulating the nucleotide-binding oligomerization domain-like receptor family pyrin domain-containing-3 (NLRP3) inflammasome (Kelley et al., 2019).

MAMs are not only implicated in the initiation of inflammatory processes but also function as a structural platform for the assembly of the NLRP3 inflammasome. In a resting state, NLRP3 is distributed in the ER membrane and cytoplasm; upon activation, it translocates to MAMs with the adaptor protein apoptotic speck protein containing a CARD (ASC), completing inflammasome assembly by sensing the ROS produced from mitochondrial damage, thereby activating interleukin 1β (IL-1β) and IL-18 to induce inflammation (Zhou et al., 2011). The unique localization of MAMs allows for efficient capture of damage signals originating from mitochondria. Furthermore, excessive mitophagy, dysregulated Ca^2+^ transport, and ER stress may result in mitochondrial impairment, the generation of ROS, exposure of mtDNA, and subsequently trigger the activation of the NLRP3 inflammasome (Missiroli et al., 2018).

In-depth analysis of the interaction mechanism between NLRP3 and MAMs will provide new targets for the treatment of inflammatory diseases.

### 2.7 Immune response

The innate immune system is an evolutionarily conserved system that functions as the first line of defense against invading microbial pathogens and other potential threats to the host. The innate immune system is modulated by a variety of germline-encoded receptors known as pattern recognition receptors (PRRs) (Paludan and Bowie, 2013). PRRs are capable of identifying pathogen-associated molecular patterns (PAMPs) that are characteristic of various microorganisms, including viruses, bacteria, parasites, and fungi. Furthermore, PRRs are involved in the detection of endogenous danger signals through the recognition of damage-associated molecular patterns (DAMPs). Certain studies indicate that MAMs may play a significant role in the immune response to viral infections.

In the antiviral innate immune defense system, mitochondrial antiviral-signaling protein (MAVS) plays a central role. MAVS is a protein located on the outer membrane of mitochondria, and also localized at the MAMs (Seth et al., 2005). As a central hub for signal transduction initiated by retinoic acid inducible gene (RIG-I)-like receptors, MAVS interacts with RIG-I to active NF-κB and interferon regulatory factor 3 (IRF3) pathways (Rajput et al., 2011). When a virus invades a cell, intracellular pattern recognition receptors (such as RIG-I-like receptors) can quickly recognize the virus’s RNA. The RIG-I-like receptor family includes RIG-I, melanoma differentiation-associated gene 5 (MDA5), and RIG-I-like receptor LGP2 (Yoneyama et al., 2005). In details, upon activation, MAVS recruits several TRAF family members (Bender et al., 2015). This recruitment leads to the phosphorylation and nuclear translocation of IRF3, as well as activation of the transcription factor NF-κB.

Interesting, the stimulator of interferon genes (STING) has been recognized as a positive modulator of RIG-I-mediated interferon-β (IFN-β) signaling pathways (Rajput et al., 2011). STING interacts with MAVS and RIG-I to form a complex to promote the phosphorylation of IRF3 and the production of IFN (Xue et al., 2022).

### 2.8 Apoptosis

Apoptosis represents one of form of programmed cell death (PCD) that is meticulously regulated to oversee cellular growth, development, and renewal. Mitochondria and the ER transmit apoptosis signals through MAMs.

In details, Fis1 forms a complex with the ER membrane protein B cell receptor-associated protein 31 (Bap31), which leads to the cleavage and generation of the pro-apoptotic protein p20Bap31 and activates caspase-8, initiating apoptosis mediated by MAMs (Liu et al., 2022).

The transport of Ca^2+^ from the ER to the mitochondria is a critical component in the process of apoptosis. B-cell lymphoma-extra-large (Bcl-xl), which is an anti-apoptotic protein belonging to the B-cell lymphoma 2 (Bcl-2) family, relocates to the MAMs under apoptotic stimuli, enhancing its interaction with IP_3_R3 to reduce cytosolic Ca^2+^ release and inhibit Ca^2+^ overload (Bonneau et al., 2013). Moreover, the anti-apoptotic function of Bcl-2 is dependent on the integrity of MAMs and its interaction with TOM20 (Lalier et al., 2021). In terms of the pro-apoptotic factor, Bax, which is a pro-apoptotic member of the Bcl-2 family, translocates to the mitochondrial membrane via MAMs, promoting the release of cytochrome c by accumulating Ca^2+^ and opening the mPTP (Lee et al., 2014).

MAMs, as a core regulatory hub of apoptosis, precisely regulate cell survival and death through the integration of multiple mechanisms, including Ca^2+^ signaling, protein complex interactions. Their functional abnormalities are closely related to various pathological processes such as neurodegenerative diseases, cardiovascular diseases, and diabetes mellitus, making them potential therapeutic targets.

## 3 The relationship between MAMs and diseases

MAMs’ proteins are integral to the onset of diseases, as they are involved in the processes that alter normal cellular signaling pathways. It has been observed that the proteins of MAMs in various pathological cell types have undergone modifications, primarily characterized by alterations in Ca^2+^ signaling and mitochondrial dynamics. These changes may influence cellular bioenergetics, consequently impacting the functions and survival of pathological cells and their sensitivity to apoptotic stimuli.

### 3.1 Cancer

#### 3.1.1 Breast cancer

**FIGURE 3:**
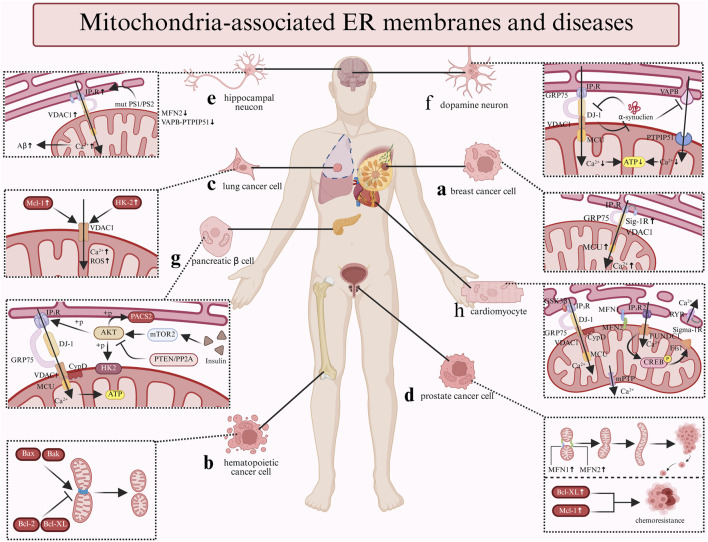
The relationship between MAM-related cellular and diseases. a: The upexpression of Sig-IR and MCU increase the Ca²^+^ flux, thereby promote breast cancer cell migration and metastasis; b: The pro-apoptotic factors Bax and Bak promote the mitochondria's fission while the anti-apoptotic factors Bcl-2 and Bcl-XL inhibit the process in hematopoietic cancer cell; c: The upexpression of Mcl-1 and HK-2 both can interact with VDAC1 to increase the Ca²^+^ flux and generate ROS in lung cancer cell; d: The upregulation of Bcl-2 family proteins (Bcl-XL/Mcl-1) correlates with chemoresistance; moreover, the fusion process which regulated by MFN1 and MFN2 facilitate prostate cancer progression; e: In hippocampal neucon, the Aβ production is promoted by the Ca²^+^ flux which controlled by the mutant PS1/PS2 and IP_3_R. Furthermore, the expression of the MFN2 and VAPB-PTPIP51 are downregulated. f: α-synuclein inhibit the VAPB-PTPIP51 complex and the IP_3_R-GRP75-VDAC-DJ-1 complex to decrease the Ca2+ flux and ATP production; meanwhile, DJ-1 also can interact with α-synuclein. g: Insulin stimulation enhances mTORC2 presence at MAMs, activating AKT. AKT phosphorylates IP_3_R to suppress Ca²^+^ release and stabilizes MAMs via PACS2/HK2 phosphorylation. These AKT-driven effects are opposed by PTEN/PP2A-mediated dephosphorylation. h: CypD and GSK3β promote the the Ca²^+^ flux via the IP_3_R-GRP75-VDAC complex; FUNDCI trigger the mitochondrial autophagy via FUNDCI-CREB-Fis1 axis while Sig-1R associates with RyR to promote ATP production. Further, MFN1/2 protect the mitochondrial integrity.

Dysregulated localization or expression of MAMs-associated proteins is closely linked to breast cancer (BC) (Figure 3a) **(** Morciano et al., 2018). Studies demonstrate that Sig-1R expression is markedly elevated in metastatic cancer cells compared to normal tissues (Gueguinou et al., 2017). Sig-1R promotes cancer cell migration by facilitating Ca^2+^ influx through the SK3 and Orai1 channels (Gueguinou et al., 2017). Notably, mitochondrial Ca^2+^ dynamics exhibit dual roles: reduced initial Ca^2+^ uptake facilitates apoptosis evasion, whereas MCU-mediated Ca^2+^ influx drives tumor proliferation and metastasis (Marchi et al., 2013). Therefore, in triple-negative breast cancer (TNBC), MCU downregulation suppresses invasiveness, mROS production, and HIF-1α expression (Tosatto et al., 2016). It is evident that the flux of Ca^2+^ in the MAMs is not solely governed by the activity of the MCU. IP_3_R3, a critical Ca^2+^ channel at MAMs, regulates tumor metabolism, as its inhibition induces autophagy-associated cell death via the Atg5 and ROS pathway, accompanied by elevated LC3-II levels (Singh et al., 2017). Notably, although MCU is not localized at MAMs, its cooperativity with IMM modulators fine-tunes mitochondrial Ca^2+^ homeostasis, a process potentially altered in cancers (Marchi et al., 2013).

#### 3.1.2 Hematopoietic cancer

Bcl-2 family proteins were initially identified within the hematopoietic and lymphoid systems (Reed, 2008) (Figure 3b). Bcl-2 family proteins regulate Ca^2+^ signaling through multiple mechanisms. Anti-apoptotic Bcl-2 binds IP_3_Rs (Davids and Letai, 2012) and targets VADC1 (Arbel and Shoshan-Barmatz, 2010) to inhibit pro-apoptotic Ca^2+^ release, blocking apoptosis in leukemia (Coustan-Smith et al., 1996). Its homolog NRH/Bcl-2L10 exerts similar effects in breast cancer, and peptides disrupting IP_3_R-Bcl-2L10 complexes suppress cancer cell proliferation (Bonneau et al., 2013). Prior research has demonstrated that Bcl-XL can facilitate mitochondrial energy production and cellular metabolism by augmenting the transfer of Ca^2+^ from the ER to the mitochondria (Williams et al., 2016). Bcl-XL displays dual functionality: its BH4 domain interacts with VDAC1 to prevent mitochondrial Ca^2+^ overload (Arbel et al., 2012) while enhancing pro-survival basal Ca^2+^ flux (Huang et al., 2013). In addition to Bcl-2 and Bcl-XL, pro-apoptotic Bax/Bak increase mitochondrial Ca^2+^ loading (Chami et al., 2004) and induce mitochondrial dysfunction (Mikhailov et al., 2003), functioning as tumor suppressors. Their expression levels directly determine cancer cellular apoptotic sensitivity.

#### 3.1.3 Lung cancer

Mcl-1 overexpression in lung cancer promotes cell migration by interacting with VDAC1 to enhance mitochondrial Ca^2+^ uptake and ROS generation (Song et al., 2005; Huang et al., 2014) (Figure 3c). Additionally, a peptide derived from VDAC1 disrupting this interaction effectively inhibit lung cancer migration (Meng et al., 2023). Hexokinase 2 (HK2), enriched at MAMs, exhibits elevated expression levels in cancer cells (Wang et al., 2024). HK2 drives the Warburg effect via VDAC1 by mitochondrial ATP utilization (Miyamoto et al., 2008). HK2 depletion restores sensitivity to agents that induce cell death and to enhance oxidative glucose metabolism (Wolf et al., 2011). Furthermore, miR-218 suppresses lung cancer proliferation by downregulating HK2 to induce apoptosis and autophagy (Wang et al., 2024). Additionally, a peptide that displaces HK2 from the mitochondria has been associated with mitochondrial Ca^2+^ overload, ultimately leading to cell death (Ciscato et al., 2020).

#### 3.1.4 Prostate cancer

In the initial stages of prostate tumor development, the tumors exhibit a reliance on androgens, rendering them susceptible to androgen ablation therapies that effectively eliminate cancer cells. Therefore, it is imperative to enhance treatment strategies to address these tumors prior to their progression to an androgen-independent pathological stage. Experiments in LNCaP cells (androgen-responsive prostate cancer) have shown that Bcl-2 is essential for both the survival of androgen-independent prostate cancer cells and the transition of prostate cancer cells from androgen-dependent to androgen-independent states (Lin et al., 2007). Moreover, upregulation of Bcl-2 family proteins (Bcl-XL/Mcl-1) correlates with chemoresistance (Maji et al., 2018) (Figure 3d). However, clinical data reveal stage-specific expression patterns: Bcl-2 is elevated in early-stage cancers but declines during progression (Ramesh et al., 2021). As mentioned in the Function section, MFN1 and MFN2 are crucial for the mitochondrial Ca^2+^ regulation and mitochondrial dynamics. The fusion-fission machinery has been implicated in both apoptosis and cancer development. Research has indicated that the promotion of mitochondrial fusion through the upregulation of MFN1 and MFN2 is linked to the advancement of prostate cancer (Philley et al., 2016). But the experimental findings supporting the tethering role of MFN2 remain controversial. Depletion of MFN2 decreases mitochondrial Ca^2+^ uptake (Wang et al., 2022), while Filadi reported that the ablation of MFN2 activates Ca^2+^ signaling that leads to cell death (Filadi et al., 2015).

Collectively, dysregulation of MAMs-associated Ca^2+^ signaling and mitochondrial dynamics represents a unifying mechanism across breast cancer, hematopoietic cancer, lung cancer, and prostate cancers, suggesting broad therapeutic potential.

### 3.2 Neurodegeneration

#### 3.2.1 Alzheimer’s disease

Alzheimer’s disease (AD) is a progressive neurodegenerative disorder characterized by cognitive decline, with core pathological hallmarks including β-amyloid (Aβ) plaques, neuronal atrophy, and neurofibrillary tangles, particularly prominent in the cortex and hippocampus (Busche and Hyman, 2020). Additional features encompass Ca^2+^ transport and cholesterol imbalance, heightened oxidative stress, mitochondrial structural abnormalities, and neuronal apoptosis (Lane et al., 2018). While the amyloid cascade hypothesis remains dominant, it fails to explain early biochemical alterations such as mitochondrial dysfunction and oxidative stress (Hardy and Higgins, 1992; Liu and Yang, 2022). Notably, MAMs dysfunction precedes Aβ plaque formation; fibroblasts from AD patients exhibit upregulated MAMs functions and increased ER-mitochondrial contacts (Area-Gomez et al., 2012). These underscore MAMs central regulatory role in AD. Hence, exploring the relationship between MAMs and AD is an extremely attractive subject.

MAMs contribute to AD pathogenesis through multiple mechanisms. In patients with AD, there is an observed upregulation of MAMs proteins, including IP_3_R and VDAC (Hedskog et al., 2013). Additionally, Aβ has been found to enhance the contacts between the ER and mitochondria and to upregulate MAMs functions in primary hippocampal neurons. Research has reported that Presenilins 1 (PS1) and presenilins 2 (PS2) localize to MAMs, where mutant PS1/PS2 bind IP_3_R to promote Ca^2+^ release and to stimulate Aβ production (Area-Gomez et al., 2009) (Figure 3e **)**. Furthermore, the decreased IP_3_R expression reduced Aβ accumulation and tau hyperphosphorylation (Shilling et al., 2014). The VDAC1-IP_3_R-GRP75 complex potentially mediates mitochondrial dysfunction and apoptosis via γ-secretase regulation (Shoshan-Barmatz et al., 2018). Furthermore, MFN2 expression is downregulated in postmortem AD brains (Leal et al., 2020), and MFN2 deficiency increases ER-mitochondrial contacts and Ca^2+^ transfer, accelerating Aβ generation (Leal et al., 2016). Moreover, the VAPB-PTPIP51 tethering complex is markedly reduced in late-stage AD cortices, correlating with disease severity (Lau et al., 2020).

Overall, these findings further support the argument that the upregulation of MAMs functions and the increased crosstalk between the mitochondria and the ER may become early pathological features of AD. We also believe that these changes are central to the pathogenesis of AD and provide important theoretical support for future research targeting MAMs proteins in AD therapies.

#### 3.2.2 Parkinson’s disease

Parkinson’s disease (PD) is the second largest neurodegenerative disease (Ben-Shlomo et al., 2024). PD is defined by Lewy bodies composed of aggregated and misfolded α-synuclein protein, with pathogenesis linked to MAMs dysfunction (Burré et al., 2010). α-Synuclein enrichment at MAMs leads to the mislocalization of the protein to mitochondria or cytosol upon point mutations, disrupting ER-mitochondrial contacts and exacerbating mitochondrial fragmentation (Guardia-Laguarta et al., 2014; Gómez-Suaga et al., 2018). Given that MAMs regulate various intracellular processes, elucidating the presence of α-synuclein in MAMs and its functional role is essential for comprehending the mechanisms by which α-synuclein operates and its deleterious effects in PD.

A recent investigation revealed that α-synuclein impairs the VAPB-PTPIP51 complex function, suppressing mitochondrial aerobic energy production (Figure 3f). Consistent with these findings, deficiencies in either VAPB or PTPIP51 have been shown to inhibit the release of synaptic vesicles and diminish the density of dendritic spines. These observations suggest that the synaptic dysfunction associated with α-synuclein in PD may be linked to a reduction in the expression of VAPB-PTPIP51(Devine and Kittler, 2018; Liu and Yang, 2022). Moreover, research indicates that mutations in the DJ-1 gene are linked to early-onset autosomal recessive PD (Bonifati et al., 2003). DJ-1 is known to exert protective effects against oxidative stress and to influence mitochondrial dynamics (Wang et al., 2012). As previously mentioned, DJ-1 is involved in the partial transport of Ca^2+^ as a constituent of the IP_3_R-GRP75-VDAC protein complex (Basso et al., 2020). Research in the context of MAMs has demonstrated that α-synuclein may disrupt the expression of the IP_3_R-GRP75-VDAC complex. Consequently, the reduced levels of DJ-1 observed in the substantia nigra of patients with PD may contribute to the disintegration of this complex, which in turn affects Ca^2+^ signaling and ATP production, ultimately impacting mitochondrial dynamics (Liu et al., 2019). Additionally, studies have indicated that DJ-1 can directly interact with both monomeric and oligomeric forms of α-synuclein, with the overexpression of DJ-1 leading to a reduction in the dimerization of α-synuclein (Zondler et al., 2014).

To a large extent, the exact pathology of PD is unknown. But the identification of disrupted ER-mitochondrial signaling transduction, which governs cellular pathways, in PD underscores the potential for a shared connection between these phenomena. However, it remains uncertain whether ER-mitochondrial signaling is enhanced or suppressed in the context of PD. Therefore, understanding MAMs and their interplays will pave the way for next-generation precision medicines for PD.

### 3.3 Diabetes mellitus

Diabetes mellitus (DM) is a disease caused by abnormal insulin secretion and action, resulting to the disturbances of glucose homeostasis (Eizirik et al., 2020). The evidence indicating that dysregulated Ca^2+^ signaling, ROS production, ER stress, and modifications in autophagy are associated with the insulin resistance and the function of β cells (Rieusset, 2015). Considering important functions played by MAMs, the dysfunction of MAMs has crucial relationship with DM.

MAMs play a central role in maintaining glucose homeostasis by regulating the localization and activity of key proteins of the insulin signaling pathway, including mTORC2, PKB, phosphatase and tensin homolog (PTEN), and protein phosphatase 2A (PP2A) (Figure 3g). Insulin stimulation promotes mTORC2 enrichment at MAMs, then activating PKB kinase (Betz et al., 2013). Activated PKB subsequently phosphorylates IP_3_R, thereby inhibiting the IP_3_R-mediated release of Ca^2+^ (Giorgi et al., 2010). Furthermore, PKB phosphorylates the resident proteins PACS2 and HK2 to preserve the structural integrity, a process that is essential for insulin signaling (Miyamoto et al., 2008; Aslan et al., 2009). Moreover, the phosphorylation effects mediated by PKB is counterbalanced by PTEN/PP2A-mediated dephosphorylation, establishing dynamic equilibrium. This process aids in the restoration of Ca^2+^ translocation, ultimately enhancing the cellular sensitivity to apoptotic stimuli (Bononi et al., 2013). The enhancement of MAMs integrity promotes insulin action, whereas disruption of the MAMs in hepatocytes results in impaired insulin signaling. Recently, research have shown that silencing the critical proteins PKB or mTORC2 which is disrupting the insulin signaling inevitably impairs the mitochondrial functions and MAMs integrity (Betz et al., 2013). The description indicates a bidirectional relationship between the integrity of MAMs and insulin signaling. It is essential to emphasize that, while there is consensus regarding the involvement of MAMs in insulin signaling, the specific mechanisms underlying this interaction remain ambiguous.

Considering that the MAMs contains various proteins integral to insulin signaling and that its structural integrity is essential for the proper function of insulin, it is not unexpected that abnormal structure and functions in MAMs are observed in the context of insulin resistance and DM (Tubbs et al., 2018). The integrity of MAMs is compromised in the livers of mice that are obese and diabetic. The liver-specific deletion of MFN2 or the inhibition of mTORC2 compromises the structural integrity of MAMs, leading to various metabolic dysregulations, such as insulin resistance and impaired glucose tolerance (Sebastián et al., 2012). In a comparable manner, the genetic deletion or pharmacological inhibition of Cyclophilin D (CypD) disrupts the integrity of the MAMs and leads to the development of hepatic insulin resistance in murine models. Conversely, the overexpression of CypD in primary hepatocytes derived from mice enhances the contacts between the mitochondria and the ER, thereby facilitating improved insulin signaling (Rieusset et al., 2016). Furthermore, the disruption of MAMs represents a significant subcellular modification that accelerates the development of muscle insulin resistance in both murine and human models.

Except for the insulin signaling and insulin resistant, MAMs also exert its effects on the modulation of the secretory mass and function of β cell. Consequently, the impaired MAMs in pancreatic β cells are regarded as a significant factor in the pathogenesis of DM. Recent findings indicated that diabetic models exhibit reduced ER-mitochondria contacts in β-cells, accompanied by ER stress and insulin secretion reduction (Thivolet et al., 2017). Moreover, modifications to MAMs proteins have implications for both the functions and mass of β cells. Mouse with depletion of IP_3_R1 exhibit disrupted ER Ca^2+^ homeostasis, increased ER stress, diminished glucose tolerance, and a reduction in β cell mass (Takeda et al., 2016). Conversely, the depletion of VDAC1 in pancreatic β cells results in an increase in ATP production, which subsequently causes depolarization of the plasma membrane, elevated cytosolic Ca^2+^ levels, and enhanced insulin secretion. This mechanism serves to safeguard β cells from elevated glucose concentrations and preserves the cell’s reductive capacity (Zhang et al., 2019).

While the study underscores the significance of MAMs in the context of DM, the regulatory mechanisms governing MAMs remain inadequately understood and necessitate additional research. The growing acknowledgment of the significance of ER-mitochondria interactions in the impairment of pancreatic islets and peripheral tissues suggests that maintaining the optimal functionality of MAMs may offer a new therapeutic strategy for diabetes mellitus.

### 3.4 Cardiovascular diseases

Recent studies have highlighted the central regulatory role of MAMs in cardiovascular pathologies. In myocardial ischemia-reperfusion injury (IRI), heart failure (HF), and diabetic cardiomyopathy (DCM), MAMs contribute to disease progression through shared mechanisms including Ca^2+^ dyshomeostasis, mitochondrial dynamics abnormalities, and energy metabolism disorders.

MAMs plays a pivotal role in myocardial damage by Ca^2+^ transport (Figure 3h). During IRI, ER-mitochondrial Ca^2+^ overload which mediated by the IP_3_Rs-GRP75-VDAC protein complex activates the mPTP via CypD-dependent pathways, triggering cardiomyocyte apoptosis (Elrod and Molkentin, 2013). Additionally, emerging evidence suggests that CypD has the capacity to interact with IP_3_Rs-GRP75-VDAC during the transfer of Ca^2+^ (Paillard et al., 2013). Research has demonstrated that pharmacological inhibition of CypD or GSK-3β effectively mitigates Ca^2+^ overload and reduced both cell death and the extent of infarct area in hearts subjected to reperfusion (Wang K. et al., 2015; Gong et al., 2021). Furthermore, the downregulation of MFN2 expression may alleviate mitochondrial Ca^2+^ overload and cellular damage by disrupting the interaction between CypD and the IP_3_R1-VDAC1 complex. This highlights the critical importance of MAMs in the regulation of the mPTP and Ca^2+^ channels, positioning them as potential therapeutic targets for the management of IRI. In HF, study has shown that there is an observed increase in the distance between the ER and mitochondria within myocardial cells, accompanied by a reduction in mitochondrial Ca^2+^ uptake (Gutiérrez et al., 2014). Hence, the disturbances in Ca^2+^ homeostasis may represent a critical characteristic of HF(Chaanine, 2021). Protein complexes including FUNDC1, IP_3_R, MFN2, and Sig-1R have been implicated in the pathogenesis of HF. Suppression of the FUNDC1-CREB-Fis1 axis causes a marked decrease in Ca^2+^ concentrations and abnormal mitochondrial fission, thereby exacerbating cardiac dysfunction and contributing to the progression of HF (Wu et al., 2017). The inhibition of Sig-1R, another critical molecule has been shown to promote autophagy in cardiomyocytes when subjected to oxidative stress (Wang et al., 2018), while Sig-1R agonists enhance Ca^2+^ signaling to improve ATP synthesis (Ortíz-Rentería et al., 2018). Additionally, Sig-1R associates with RyR to form another complex, which diminishes Ca^2+^ leakage, mitigates myocardial hypertrophy, and enhances ATP synthesis (Tagashira et al., 2013). In DCM, under conditions of elevated glucose levels, the inactivation of AMPK triggers the activation of FUNDC1, promoting the formation of MAMs and abnormal mitochondrial autophagy. Concurrently, FUNDC1 interacts with IP_3_R2, obstructing its ubiquitination, enhancing Ca^2+^ release into mitochondria, thereby exacerbating mitochondrial dysfunction and adversely affecting myocardial function (Wu S. et al., 2019). The genetic deletion of FUNDC1 markedly inhibits the formation of MAMs induced by high glucose levels, consequently inhibiting regulatory pathways and reversing the progression of DCM (Wu S. et al., 2019). Consequently, the deregulation of FUNDC1-associated MAMs may offer a novel therapeutic approach for the management of DCM.

Mitochondrial dynamics which regulate the CVDs represents another core MAMs mechanism. MFN1/2-mediated fusion and DRP1-controlled fission processes are crucial for myocardial protection. Prior studies have indicated that excessive mitochondrial fission within myocardial cells is recognized as a significant contributor to the onset of IRI (Sharp et al., 2014). MFN2 overexpression or mdivi-1 treatment preserves mitochondrial network integrity to reduce sensitivity to mPTP opening and the cellular mortality following IRI (Ong et al., 2010). Furthermore, the decrease in the expression levels of MFN1 and MFN2 has been shown to mitigate IRI in mouse myocardial tissue and to diminish the extent of infarction (Hall et al., 2016). In HF, study have illustrated that the reduction of MFN2 expression leads to mitochondrial fragmentation in both *in vivo* and *in vitro* models of heart failure (Liu J. et al., 2021; Xu et al., 2021). Furthermore, emerging evidence suggests that the upregulation of MFN2 not only mitigated the production of ROS and restored mitochondrial membrane potential, but also inhibited cardiomyocyte hypertrophy and the associated pro-hypertrophic phenotype (Sun et al., 2019).

These findings establish MAMs as a convergent pathological hub integrating Ca^2+^ signaling, mitochondrial dynamics, and energy metabolism across cardiovascular diseases. Targeting key MAMs components like MFN2, FUNDC1, and IP_3_R provides a theoretical foundation for developing novel therapeutic strategies with cross-disease applicability.

Despite the diverse pathological phenotypes of various diseases, the dysfunction of MAMs is predominantly linked to four fundamental regulatory functions: the imbalance of Ca^2+^ homeostasis, abnormal mitochondrial dynamics, metabolic reprogramming, and the dysregulation of stress responses. Notably, Ca^2+^ transport, a critical function of MAMs, demonstrates an imbalance in Ca^2+^ homeostasis in both breast cancer cells and dopamine neurons. However, the downstream consequences of this imbalance lead to distinct pathological outcomes: an increase in Ca^2+^ flux contributes to apoptosis resistance in cancer cells, whereas a decrease in Ca^2+^ flux is associated with abnormal energy metabolism. This phenomenon of “shared pathways-differentiated effects” indicates that MAMs serve as essential regulatory hubs across various diseases, necessitating targeted interventions that consider the specific tissue microenvironment. Future investigations should aim to further clarify the spatiotemporal dynamics of MAMs protein complex to uncover its molecular plasticity in different disease contexts.

## 4 The different therapeutic targets that act through the mechanisms associated with MAMs

Recent literature indicates that contact sites between the ER and mitochondria may be integral to various stages of disease progression. However, research focusing on the key targetable components located within MAMs remains insufficiently comprehensive. In this context, we aim to explore novel pharmacological agents that improve drug utility and sensitivity through the modulation of signaling pathways that connect the mitochondria and ER. Additionally, we seek to identify new potential therapeutic targets within these associated signaling pathways for treatment. Considering the distinctive roles of MAMs in Ca^2+^ transport, mitochondrial dynamics, autophagy, and metabolism, which render them valuable as both diagnostic markers and therapeutic targets, we will subsequently engage in a discussion of these four domains (Table 2).

**TABLE 2:** Overview the different therapeutic targets that act through the mechanisms associated with MAMs.

Principal function	Target protein	Treatment	Mechanism of action at MAMs	Related diseases	References
Ca^2+^ signaling	Bcl-2	ABT737	Decreasing Ca^2+^ signaling, reducing ERS and increasing MAMs contacts by targeting the BH3 domain of Bcl-2	OC	Xie et al. (2016), Xu et al. (2017)
		TAT-IDPs	Increasing IP_3_R-mediated Ca^2+^ release by targeting the BH4 domain of Bcl-2	OC	Xie et al. (2017)
		BIRD-2	Increasing IP_3_R-mediated Ca^2+^ release by targeting the BH4 domain of Bcl-2, trigger Ca^2+^ overload and open mPTP	CLL; DLBCL	Kerkhofs et al. (2021)
	GRP75	Knockdown of GRP75	Diminishing MAM integrity by GRP75-deficiency reduces ER-mitochondria Ca^2+^ transfer and accelerates mitochondrial dysfunction; Increasing cisplatin-induced cancer cell death	OC	Li et al. (2022a)
	VDAC1	Knockdown of VDAC1	Downregulating the expression of VDAC1 induces apoptosis	Cancer	Shoshan-Barmatz et al. (2017)
		Peptide	Interfering with the interaction between VDAC1 and anti-apoptotic factors (e.g., Bcl-2, Bcl-xl, HK)	B-CLL	Prezma et al. (2013)
	IP_3_R	IP_3_R-MT	Sensitizing tumour cells with low or no PTEN expression to photodynamic therapy based on Ca^2+^-dependent cytotoxicity	PC	Kuchay et al. (2017)
		Panaxydol	Activating IP_3_R leads to the release of Ca^2+^ from the ER, which subsequently activates JNK signaling pathway, results in ERS and triggers apoptotic	PC and RCC	Kim et al. (2016), Martucciello et al. (2020)
		2-APB	Increasing the level of PGC-1α and TFAM via the inhibition of IP_3_R	PD	Piccinin et al. (2021)
		Xestospongin C	Mitigating Ca^2+^ overload by inhibiting IP_3_R	AD	Wang et al. (2019)
		β-Carotene	Mitigating intracellular Ca^2+^ levels by specifically interacting with the IP_3_R-GRP75-VDAC1-MCU complex, facilitating the repair of oxidative damage	Mastitis	Meng et al. (2023)
	NK-1R	Aprepitant and SR140333	Stimulating oxidative stress and increased mitochondrial ROS induce apoptosis in myeloid leukemia cells	AML	Ge et al. (2019)
	HK2	Peptide	Displacing of HK2 from MAMs with a selective peptide trigger mitochondrial Ca^2+^ overload	B-CLL; BC; CC	Ciscato et al. (2020)
		miR-218	Targeting HK2 and LDHA to inhibit tumor growth and angiogenesis	Cancer	Wang et al. (2024)
	Sigma-1R	SKF10047	Increasing ER-mitochondria contacts and IP_3_R-GRP75-VDAC Ca^2+^ transport complexes expression	Hypertension	Ooi et al. (2021)
		PRE-084	Recovering of Ca^2+^ transfer and mitochondrial respiration *in vitro*, which subsequently rectify the associated elevation in autophagy and mitophagy	WS	Crouzier et al. (2022)
		CGI1746	Diminishing the formation of MAMs and impeding the transfer of Ca^2+^ from the ER to the mitochondria	Acute kidney injury	Zhang et al. (2024)
	MFN2	Overexpression of MFN2	Engaging with SERCA2 to augment the contacts between MAMs and to improve T cell cytotoxicity; elevating levels of ROS and inducing apoptosis	LC; HCC	Rehman et al. (2012), Yang et al. (2023)
		miR-761	Inhibiting targeted MFN2 to increase MFN2 levels; suppressing the increase of intracellular ROS to inhibit tumor growth and metastasis	OC	Zhou et al. (2016)
Dynamics	DRP1	Mdivi-1	Inhibiting DRP1	AD; PD; IRI	Dai et al. (2020)
		P110	Inhibiting GTPase activity of DRP1 and its interaction with Fis1	PD; HD	Guo et al. (2013), Qi et al. (2013)
		Dynasore	Inhibiting GTPase activity of Dynamin 1, Dynamin 2, DRP1	AD; CVD	Ong et al. (2015), Medala et al. (2021)
		Knockdown or siRNA of DRP1	Inhibiting DRP1 and promoting mitochondrial fusion	Cancer	Lin et al. (2020)
	MFN2	HO-1	Upregulating MFN1/2 expression	Cardiomyopathy	Hull et al. (2016)
		Melatonin	Activating the Notch1/MFN2 signaling pathway; upregulating MFN2 expression	IRI	Pei et al. (2016)
		M1	Promoting the mitochondrial fusion	IRI	Maneechote et al. (2019)
		S_3_	Deubiquitinating MFN1/2; augmenting the activity of MFN1/2	IRI	Maneechote et al. (2019)
		Overexpression of MFN2	Promoting fusion by increasing expression of MFN2	Cancer; Diabetes	Yue et al. (2014)
	OPA1	Punicalagin	Stimulating OPA1 to promote mitochondrial fusion	Diabetes	Fu et al. (2021)
		κ-opioid receptor	Stimulating OPA1 to promote mitochondrial fusion	IRI	Wang et al. (2020)
		Paeonol	Activating the STAT3-CK2α signaling pathway to stimulate OPA1	Diabetes	Liu et al. (2021a)
		Overexpression of OPA1	Promoting mitochondrial fusion by enhancing expression of OPA1	AD; CVD	Liu et al. (2020b)
Autophagy	FUNDC1	Melatonin	Blocking (PPAR-γ)/FUNDC1/mitochondrial autophagy pathway; inhibiting mitochondrial ATP production and platelet activation	IRI	Zhou et al. (2017)
		Knockdown or siRNA of CK2α	Promoting the upregulation of FUNDC1-dependent mitophagy	IRI	Zhou et al. (2018)
		siRNA of FUNDC1	Increasing BMl1 expression by promoting Ca^2+^ cytosol influx, nuclear translocation and activity of NFATC1	BC	Wu et al. (2019a)
	PML	Overexpression of PML	Reducing ER-mitochondrial Ca^2+^ transfer, mitochondrial respiration, and ATP production, stimulating autophagy via the AMPK/mTOR/Ulk1 pathway	Cancer	Missiroli et al. (2016)
	DJ-1	Overexpression of DJ-1	Increasing the IP_3_R-GRP75-VDAC1 complex inhibits the accumulation of IP_3_R within the MAMs and promotes the clearance of α-synuclein by microglia	PD	Nash et al. (2017), Liu et al. (2019)
Metabolic	TMX1	Knockdown or siRNA of TMX1	Affecting ER-mitochondrial Ca^2+^ transfer and mitochondrial bioenergetics by inhibiting SERCA2b	Cancer	Raturi et al. (2016)
	GSK3β	Inhibition of GSK3β	Promoting the recruitment of HK-I to the VDAC, influencing mitochondrial respiration and thus supporting the metabolic reprogramming required for memory CD8^+^ T cells	Leukaemia	Bantug et al. (2018)

### 4.1 Ca^2+^ transport

Various approaches can be employed to influence cellular functions by enhancing or attenuating Ca^2+^ transport through the modulation of MAMs proteins.

Currently, the predominant factor for targeting Ca^2+^ transport is Bcl-2. Bcl-2, as an important anti-apoptotic factor in the intracellular, could yield good results if it can be targeted to inhibit its functions. Research has demonstrated that the overexpression of Bcl-2 diminishes the growth inhibition and apoptotic effects induced by cisplatin in human ovarian cancer (OC) cells (Xu et al., 2017). The TAT-fused inositol 1,4,5-trisphosphate receptor-derived peptide (TAT-IDP_S_), which specifically interacts with the BH4 domain of Bcl-2, has been shown to enhance cisplatin-induced Ca^2+^ flux from the ER into both the mitochondria and cytosol (Xie et al., 2017). The finding suggests that TAT-IDP_S_ has the potential to augment the cytotoxic effects of cisplatin on OC cells through the facilitation of ER Ca^2+^ release (Xie et al., 2017). And other therapeutic target is BIRD-2, which also targets the BH4 domain of Bcl-2, has defined that causing the Ca^2+^ overload and opening the mPTP, resulting the death of the diffuse large b-cell lymphoma (DLBCL) and chronic lymphocytic leukemia (CLL) cell (Kerkhofs et al., 2021). Another MAMs target for treating OC is GRP75. GRP75 deficiency perturbed MAM integrity, reduced ER-mitochondrial Ca^2+^ transfer, exacerbated CP-induced mitochondrial dysfunction, and triggered marked ROS elevation with enhanced apoptosis in OC cells (Li J. et al., 2022). An additional element of the Ca^2+^ transport complex, VDAC1, has the potential to function as both a therapeutic target and a biomarker for BC, given that the overexpression was identified in a prior investigation (Shoshan-Barmatz et al., 2017). So far, there are two therapeutic methods based on the VDAC1. Initially, one strategy involves the downregulation of overexpressed VDAC1 through the application of RNA interference techniques, such as shRNA and siRNA, which ultimately leads to the induction of apoptosis in cancer cells. Furthermore, considering that HK, Bcl-2, and Bcl-xl exert their anti-apoptotic effects by interacting with VDAC1, there exist VDAC1-derived peptides specifically engineered to disrupt these interactions, thereby facilitating the induction of apoptosis (Prezma et al., 2013; Shoshan-Barmatz et al., 2017). As for the basic element of the Ca^2+^ transport complex, Kuchay et al. proposed that inhibition of IP_3_R3 degradation in PTEN-dysregulated cancers could be a novel treatment strategy. This study demonstrated that PTEN competes with FBXL2 for binding to IP_3_R3, thereby influencing its degradation. The findings indicate that a non-degradable IP_3_R3 mutant enhances the sensitivity of PTEN-low/absent tumors to Ca^2+^-dependent photodynamic therapy (Kuchay et al., 2017). Furthermore, panaxydol was shown to induce Ca^2+^ release via IP_3_R, activating the JNK signaling pathway and triggering PERK-dependent ER stress (Kim et al., 2016; Martucciello et al., 2020). As for the neurodegenerative diseases, the high-concentration IP_3_R inhibitor 2-APB upregulated PGC-1α and TFAM expression, ameliorating motor and mitochondrial impairments in Parkinson’s disease murine models (Piccinin et al., 2021). Similarly, Xestospongin C alleviated Aβ-induced Ca^2+^ overload in hippocampal neurons and improved cognitive function in APP/PS1 mice (Wang et al., 2019). Additionally, β-carotene reduced intracellular Ca^2+^ levels by targeting the IP_3_R-GRP75-VDAC1-MCU signaling axis, thereby repairing mitochondrial oxidative damage (Meng et al., 2023). Moreover, inhibition of the neurokinin-1 receptor (NK-1R) has emerged as a promising therapeutic target for the development of novel pharmacological agents. This inhibition has been demonstrated to induce oxidative stress and elevate mitochondrial ROS levels through a rapid Ca^2+^ flux from the ER to the mitochondria. This process subsequently leads to the induction of apoptosis in myeloid leukemia cells, both *in vitro* and *in vivo*. In recent study, two NK-1R antagonists, aprepitant and SR140333 may work synergistically with conventional chemotherapies to treat human myeloid leukemia (Ge et al., 2019). The removal of HK2 from the MAMs through the application of a specific peptide induces an overload of Ca^2+^ within the mitochondria. This event subsequently activates calpain in a Ca^2+^-dependent manner, which results in mitochondrial depolarization and ultimately leading to cell death in chronic lymphocytic leukemia B cells, as well as in breast and colon cancer cells (Ciscato et al., 2020). Furthermore, subsequent research demonstrated that miR-218 suppresses tumor growth and angiogenesis through the downregulation of HK2 and LDHA expression *in vivo* (Wang et al., 2024). There are a lot of the Sig-1R agonists and antagonists have received positive responses in clinical trials. SKF10047 has been demonstrated to activate the Sig-1R, leading to a substantial increase in the protein expression of the components involved in the IP_3_R-GRP75-VDAC transport system, as well as an enhancement of Ca^2+^ transport associated with MAMs (Ooi et al., 2021). Consequently, SKF10047 proves to be advantageous in inhibiting NLRP3 activation and the pM1 polarization of microglia in rats subjected to stress-induced hypertension, thereby mitigating the occurrence of neuroinflammation. Comparably, the Sig-1R agonist PRE-084 has been shown to restore Ca^2+^ transfer in MAMs and enhance mitochondrial respiration. This compound may serve as a therapeutic agent for alleviating symptoms related to Wolfram syndrome in preclinical models (Crouzier et al., 2022). In contrast, CGI1746 acts as an inhibitor of Sig-1R, which leads to a reduction in the formation of MAMs and hinders the transfer of Ca^2+^ from the ER to the mitochondria. As a result, CGI1746 plays a role in alleviating mitochondrial Ca^2+^ overload and provides protective effects against cellular damage associated with ferroptosis (Zhang et al., 2024). Furthermore, research has demonstrated that overexpression of MFN2 promotes the interaction between the mitochondria and the ER by binding to the ER-resident Ca^2+^-ATPase SERCA2 in tumor-infiltrating CD8^+^ T cells (Yang et al., 2023). Enhancing the interaction through the upregulation of MFN2 in CD8^+^ T cells has been shown to augment the effectiveness of cancer immunotherapy. On the other hands, the implementation of effective strategies aimed at restoring mitochondrial network formation in lung cancer cells and hepatocellular cancer cells through the overexpression of MFN2 has been associated with a significant decrease in cancer cell proliferation and an enhancement of spontaneous apoptosis (Rehman et al., 2012; Wang W. et al., 2015). Notably, the upregulation of MFN2 can be achieved through the miR-761, and then disrupts mitochondrial function and significantly inhibits tumor growth and metastasis in both *in vivo* and *in vitro* (Zhou et al., 2016).

Ca^2+^ signaling, regulated by the interactions between the ER and mitochondria via MAMs, plays a key role in the treatment of various diseases. Therapeutic strategies that focus on the modulation of Ca^2+^ signaling pathways offer multifaceted intervention methods for cancer, neurodegenerative disorders, and metabolic diseases, by precisely regulating the functions of MAMs.

### 4.2 Dynamics

MAMs serve as physical platforms facilitating the processes of mitochondrial fission and fusion. Critical proteins located on these platforms, including DRP1, MFN1/2, and OPA1, play essential roles in the regulation of mitochondrial dynamics. Research shows that restoring mitochondrial dynamics through various methods, including gene therapy that alters gene expression to influence mitochondrial dynamics processes, as well as chemotherapy targeting various mechanisms essential for mitochondrial fission and fusion, can improve tissue functionality and prolong lifespan in animal models (Jüschke et al., 2021).

As previously noted, DRP1 is a dynamin-related protein that plays a critical role in mitochondrial fission (Smirnova et al., 2001). Upon activation, DRP1 accumulates to the OMM, leading the constriction and subsequent division of the mitochondria. Fission inhibitors have been engineered to reduce the levels of DRP1, thereby inhibiting mitochondrial division and facilitating mitochondrial fusion. These inhibitors demonstrate potential in mitigating the onset and progression of various diseases. Mdivi-1 is the first identified inhibitor that specifically targets proteins involved in mitochondrial division. It selectively impedes the function of DRP1 by primarily interacting with an orthosteric domain (Cassidy-Stone et al., 2008). Multiple studies have illustrated that implementation of Mdivi-1 significantly enhances behavioral outcomes and diminishes neurodegeneration in animal models of AD and PD (Oliver and Reddy, 2019). Furthermore, Mdivi-1 mitigates excessive cellular necrosis and reduces the infarct area in cardiomyocytes during IRI (Maneechote et al., 2020). And then, it also has demonstrated efficacy in inhibiting the proliferation and dissemination of malignant cells, reversing resistance to oncological treatments, and augmenting MHC-I expression in mouse tumor models (Dai et al., 2020; Lei et al., 2022). As for P110, a small peptide that inhibits mitochondrial division, has been shown to reduce both mitochondrial division and necrosis in neurons obtained from patients with Huntington’s disease (HD) and PD (Guo et al., 2013; Qi et al., 2013). Additionally, research has shown that Dynasore mitigates cardiac pathology and diminishes neuronal injury associated with degenerative diseases by inhibiting excessive mitochondrial fission (Ong et al., 2015; Medala et al., 2021). In addition to employing compounds to target MAMs proteins, modifications in gene expression can be achieved via genetic pathways, thereby regulating mitochondrial dynamics in cell. The inhibition of DRP1 leads to compromised mitochondrial autophagy and heightened apoptosis in HCC cells subjected to hypoxic conditions. This phenomenon is correlated with a decrease in mitochondrial membrane potential, alongside the release of cytochrome c and apoptosis-inducing factor. Thus, it will be a potential therapy for the HCC patients by down-regulating DRP1 (Lin et al., 2020).

MFN1 and MFN2 (Chen et al., 2003; Detmer and Chan, 2007) are situated on the OMM, while OPA1 (Al Ojaimi et al., 2022) is found on the IMM. These proteins are crucial for the mechanism of mitochondrial fusion. In mouse models exhibiting dilated cardiomyopathy, the overexpression of heme oxygenase-1 (HO-1) was found to enhance mitochondrial fusion through the upregulation of MFN1 and MFN2 expression levels (Hull et al., 2016). Serves as an additional agent, Melatonin promotes mitochondrial fusion via stimulating the Notch1/MFN2 signaling pathway, consequently increases the expression levels of MFN2 (Pei et al., 2016). Furthermore, Hydrazone M1 is a compound that facilitates mitochondrial fusion by reinstating the fusion process in MEFs (murine embryonic fibroblasts) that are deficient in MFN1 and MFN2 (Maneechote et al., 2019). It is noteworthy that 15-oxospiramilactone (S3) has the capacity to enhance ubiquitination without leading to the degradation of MFN1/2 by USP30. This process ultimately increases the activity of MFN1/2 and initiates mitochondrial fusion (Yue et al., 2014). As for OPA1, there are also several options. In terms of promoting OPA1-STAT3 pathway, punicalagin (Fu et al., 2021) and κ-opioid receptor (Wang et al., 2020) are two compounds which can promote mitochondrial fusion by enhancing the expression of OPA1. Besides, paeonol is a compound that promotes mitochondrial fusion via the OPA1 mechanism by activating the STAT3-CK2α signaling pathway in the context of diabetic cardiomyopathy (Liu C. et al., 2021). As previously indicated, there are some methods to regulate the mitochondrial dynamics by genetic pathways. On the one hand, the overexpression of OPA1 restores mitochondrial quality control and preserves the functionality of cardiomyocytes in a model of hypoxia-induced cardiomyocyte injury (Liu Y.-N. et al., 2020). On the other hand, MFN2 overexpression exerts positive function in lung cancer cells (Rehman et al., 2012) and HepG2 cells (Gao et al., 2018).

Dysregulation of MAMs’ proteins is significantly associated with the development of these diseases. Currently, targeting the related proteins on the MAMs offers a therapeutic approach for various diseases by precisely regulating the fission-fusion equilibrium via modulating DRP1, MFN1/2, and OPA1. Consequently, the regulation of MAMs to restore cellular functions may represent a novel therapeutic approach for the management of these diseases.

### 4.3 Autophagy

Mitophagy maintains dynamic homeostasis of cells through targeting dysfunctional mitochondria for timely clearance and recycling. MAMs serve as the critical platform coordinating this process, as they are essential for autophagosome formation by providing membrane contact sites for autophagy initiation. Dysfunction in MAMs is implicated in the molecular mechanisms underlying the initiation and progression of various human diseases, therefore targeting MAMs’ mitophagy-related proteins is a viable way.

With the MAMs microenvironment, FUNDC1 is a mitochondrial receptor situated in the OMM that governs the process of mitophagy. FUNDC1-dependent mitophagy is crucial for preserving the quality and functionality of mitochondria in platelets, thereby resulting platelet activation. This process contributes to decreased blood oxygen levels, potentially resulting in myocardial infarction (Zhang et al., 2017). Consequently, the inhibition of mitophagy during the acute phase of I/R may mitigate myocardial injury. And the modulation of the peroxisome proliferator-activated receptor gamma (PPAR-γ)/FUNDC1/mitophagy pathways may serve as a viable strategy for mitigating platelet hyperactivity and IRI (Zhou et al., 2017). CK2α, an inhibitor of FUNDC1, exacerbates mitochondrial apoptosis during cardiac IRI by inactivating protective mitophagy. In contrast, the lack of CK2α has been demonstrated to maintain cardiac structure and function during I/R stress by facilitating the upregulation of FUNDC1-dependent mitophagy (Zhou et al., 2018). Moreover, studies have indicated that FUNDC1 is essential for the regulation of mitochondrial division at MAMs interfaces. It facilitates the expression of the cancer stem cell marker BMI1 (polycomb ring finger oncogene) by enhancing the influx of Ca^2+^ into the cytosol and promoting the nuclear translocation and activity of nuclear factor of activated T cell 1 (NFATC1). This mechanism subsequently stimulates the proliferation, migration, and invasion of breast cancer cells, and it also functions as a predictor of poor outcomes in patients diagnosed with BC (Wu L. et al., 2019). This indicates that the inhibition of FUNDC1 or its associated pathway may represent a potential therapeutic target for the treatment of BC. MAMs further regulate autophagy through other resident proteins. As previously noted, DJ-1, which is localized to MAMs exhibits a significant relationship with α-syn. And the absence of DJ-1 results in the accumulation of α-syn. Consequently, studies have demonstrated that the overexpression of DJ-1 can enhance the autophagic capacity of microglia, thereby improving their ability to eliminate α-syn (Gao et al., 2012). This mechanism may potentially mitigate or even reverse the symptoms experienced by patients with PD. Furthermore, research has demonstrated that the loss of PML protein leads to a decline in Ca^2+^ transport, mitochondrial respiration, and ATP synthesis. This decrease subsequently initiates autophagy via the activation of the AMPK/mTOR/Ulk1 signaling pathway. Consequently, the loss of PML from the MAMs not only heightens resistance to apoptotic stimuli but also facilitates adaptation to metabolic stress and cellular damage induced by anticancer therapies (Missiroli et al., 2016). This research presents evidence that the inhibition of autophagy, when used in conjunction with chemotherapy agents like 5-fluorouracil (5-FU), may improve the sensitivity of cancer cells to chemotherapy. Consequently, this approach could contribute to the development of anticancer strategies that simultaneously modulate both autophagy and apoptosis in cancer cells.

Collectively, these studies collectively assessed the viability of targeting proteins involved in the regulation of autophagy at the level of MAMs for therapeutic interventions in various diseases, thereby underscoring their considerable potential as therapeutic targets.

### 4.4 Metabolic

MAMs proteins which regulate the metabolic reprogramming in cells suggests that targeting metabolic pathways at the contact sites between the ER and mitochondria presents novel opportunities for disease treatment. For example, Raturi et al. demonstrated that the tumor suppressor TMX1 interacts with SERCA2b within the MAMs, promoting mitochondrial ATP synthesis and triggering apoptosis in cancer cells by influencing the ER-mitochondrial contacts and the intracellular Ca^2+^ flux between the ER and mitochondria (Raturi et al., 2016). Therefore, therapeutic strategies targeting TMX1 may further enhance cancer cell death through mitochondrial metabolic regulation. Furthermore, it has been observed that cell metabolism, regulated by MAMs, not only alters the properties of tumor cells but may also influence the function of tumor-associated immune cells. In human peripheral blood leukemic T cell line, the rapid activation of PKB by mTORC2 results in the inhibition of GSK3β which situated at MAMs. This process facilitates mitochondrial respiration by promoting metabolite flux into mitochondria. This modulation is essential for the metabolic reprogramming required for memory CD8^+^ T cells to enhance therapeutic outcomes (Bantug et al., 2018).

## 5 Conclusion and perspectives

In recent decades, there has been an increasing focus on the mechanisms of intracellular communication occurring at close proximity between organelles. The ER and mitochondria are essential organelles within mammalian cells, serving as critical sites for protein synthesis and energy production. MAMs function as a critical communication interface between the ER and mitochondria. It plays a vital role in maintaining Ca^2+^ homeostasis, facilitating lipid synthesis, transportation, and metabolism, regulating autophagy and cellular stress responses, as well as overseeing the morphology and functionality of both the ER and mitochondria. It has been demonstrated that the crucial intracellular signaling platforms formed by MAMs undergo alteration during the development of a range of diseases, which in turn contributes to the abnormalities of cells. Future studies should prioritize elucidating the molecular mechanisms governing MAMs plasticity in disease contexts, particularly in terms of elucidating their role in the progression of diseases including cancer, neurodegeneration, metabolic syndrome and cardiovascular diseases.

As described in the review, previous studies have investigated the potential of targeting ER-mitochondrial contacts for therapeutic purposes. However, the majority of these studies are constrained to Ca^2+^ signaling-mediated apoptosis pathways. Recent research findings have increasingly indicated that the dynamics of mitochondria, autophagy, and metabolic signaling pathways associated with MAMs hold considerable promise for the identification of novel therapeutic targets. Consequently, it is plausible to propose that future pharmacological development aimed at these signaling pathways on MAMs may yield innovative therapeutic strategies.

While various metabolic and signaling pathways associated with MAMs have been elucidated, as well as their involvement in disease mechanisms, numerous questions remain to be addressed. For example, it is essential to examine whether MAMs exhibit consistent structural and functional characteristics across various tissues affected by different diseases. If discrepancies are identified, it is crucial to elucidate the fundamental mechanisms underlying their functionality for precise treatment. Secondly, it is essential to investigate the impact of external risk factors such as ER stress, hypoxia, viral infections, and nutritional deprivation on MAMs. To what extent do these factors play a role in the pathogenesis of diseases via MAMs? Thirdly, given their significance as a signaling platform, can MAMs serve a preventive function by acting as early progression and prognostic indicators of diseases? Finally, how to create pharmacological agents that specifically target MAMs for future investigative studies?

In conclusion, it is crucial to comprehend the functions of MAMs in the context of diseases by clarifying the molecular mechanisms that govern the regulation of MAMs. The advancement of novel methodologies and technologies in molecular and cellular biology has positioned MAMs, as a recently recognized subcellular organelle, as a promising target for therapeutic interventions in various diseases.

## Glossary

AD: Alzheimer’s disease
ATG: Autophagy-related genes
ATF6: Activating transcription factor 6
Bap31: B cell receptor-associated protein 31
BC: Breast cancer
Bcl-2: B-cell lymphoma-2
Bcl-xl: B-cell lymphoma-extra-large
CVDs: Cardiovascular diseases
CypD: Cyclophilin D
DAMPs: Damage-associated molecular patterns
DCM: Diabetic cardiomyopathy
DM: Diabetes mellitus
DRP1: Dynamin-related protein 1
ER: Endoplasmic reticulum
ERO1: Endoplasmic reticulum oxidoreductin 1-α
Fis1: Fission protein 1
FUNDC1: FUN14 domain containing 1
GRP75: Glucose-regulated protein 75 kDa
HD: Huntington’s disease
HF: Heart failure
HK2: Hexokinase 2
HO-1: Heme oxygenase-1
**H**_**2**_ **O**_**2**_: Hydrogen peroxide
IFN-β: Interferon-β
IL-1β: Interleukin 1β
IMM: Inner mitochondrial membrane
IP3R: Inositol 1,4,5-trisphosphate receptor
IRE1: Inositol-requiring enzyme 1
IRF3: Interferon regulatory factor 3
IRI: Ischemia-reperfusion injury
MAMs: Mitochondria-associated ER membranes
MAVS: Mitochondrial antiviral-signaling protein
MCU: Mitochondrial calcium uniporter
MDA5: Melanoma differentiation-associated gene 5
MEFs: Murine embryonic fibroblasts
MFN1/2: Mitofusin 1/2
MFF: Mitochondrial fission factor
mPTP: Mitochondrial permeability transition pore
mtDNA: Mitochondrial DNA
NFATC1: Nuclear factor of activated T-cells cytoplasmic 1
NLRP3: Nucleotide-binding oligomerization domain-like receptor family pyrin domain-containing 3
NK-1R: Neurokinin-1 receptor
OC: Ovarian cancer
OMM: Outer mitochondrial membrane
OPA1: Optic atrophy type 1
PA: Phosphatidic acid
PAMPs: Pathogen-associated molecular patterns
PC: Phosphatidylcholine
PCD: Programmed cell death
PD: Parkinson’s disease
PE: Phosphatidylethanolamine
PERK: Protein kinase R-like endoplasmic reticulum kinase
PISD: Phosphatidylserine decarboxylase
PKB: Protein kinase B
PRRs: Pattern recognition receptors
PS: Phosphatidylserine
PSS1 and PSS2: Phosphatidylserine synthases 1 and 2
PTEN: Phosphatase and tensin homolog
PTPIP51: Protein tyrosine phosphatase interacting protein 51
ROS: Reactive oxygen species
Sig-1R: Sigma-1 receptor
STING: Stimulator of interferon genes
Syn17: SNARE protein syntaxin 17
TCA: Tricarboxylic acid cycle
TNBC: Triple-negative breast cancer
UPR: Unfolded protein response
VAPs: Vesicle-associated membrane protein-associated proteins
VDAC1: Voltage-dependent anion channel 1
5-FU: 5-fluorouracil
